# Who would benefit from open abdomen in severe acute pancreatitis?—a matched case-control study

**DOI:** 10.1186/s13017-021-00376-x

**Published:** 2021-06-10

**Authors:** Henrik Leonard Husu, Ari Kalevi Leppäniemi, Panu Juhani Mentula

**Affiliations:** Department of Gastrointestinal Surgery, University of Helsinki and Helsinki University Hospital, P.O. Box 340, FI-00029 HUS Helsinki, Finland

**Keywords:** Abdominal compartment syndrome, Abdominal vac, Acute pancreatitis, Circulatory failure, Intra-abdominal hypertension, ICU, Laparostomy, Multiple organ failure, Necrotizing pancreatitis, Negative-pressure wound therapy, NPWT, Open abdomen, Organ failure, Renal failure, Severe acute pancreatitis, VAWCM, Vacuum-assisted wound closure, Mesh-mediated fascial traction

## Abstract

**Background:**

Selection of patients for open abdomen (OA) treatment in severe acute pancreatitis (SAP) is challenging. Treatment related morbidity and risk of adverse events are high; however, refractory abdominal compartment syndrome (ACS) is potentially lethal. Factors influencing the decision to initiate OA treatment are clinically important. We aimed to study these factors to help understand what influences the selection of patients for OA treatment in SAP.

**Methods:**

A single center study of patients with SAP that underwent OA treatment compared with conservatively treated matched controls.

**Results:**

Within study period, 47 patients treated with OA were matched in a 1:1 fashion with conservatively treated control patients. Urinary output under 20 ml/h (OR 5.0 95% CI 1.8-13.7) and ACS (OR 4.6 95% CI 1.4-15.2) independently associated with OA treatment. Patients with OA treatment had significantly more often visceral ischemia (34%) than controls (6%), P = 0.002. Mortality among patients with visceral ischemia was 63%. Clinically meaningful parameters predicting developing ischemia were not found. OA treatment associated with higher overall 90-day mortality rate (43% vs 17%, P = 0.012) and increased need for necrosectomy (55% vs 21%, P = 0.001). Delayed primary fascial closure was achieved in 33 (97%) patients that survived past OA treatment.

**Conclusion:**

Decreased urine output and ACS were independently associated with the choice of OA treatment in patients with SAP. Underlying visceral ischemia was strikingly common in patients undergoing OA treatment, but predicting ischemia in these patients seems difficult.

**Supplementary Information:**

The online version contains supplementary material available at 10.1186/s13017-021-00376-x.

## Background

Intra-abdominal hypertension (IAH) affects most patients with severe acute pancreatitis (SAP). In acute pancreatitis, IAH correlates with significant mortality, and escalation to abdominal compartment syndrome (ACS) leads to dire outcomes [1, 2]. Disproportionate fluid resuscitation might aggravate IAH in SAP, leading ultimately to ACS and increased risk of bowel ischemia [3]. Conservative management is the corner stone treatment of elevated intra-abdominal pressure (IAP) [4]. When ACS is refractory and resistant to conservative management, surgical decompression via laparostomy with following open abdomen (OA) treatment is considered [4]. Laparostomy efficiently diminishes IAP in SAP, but whether or not it attenuates evolving ischemia or reverses existing organ failures in ACS is unknown [3]. Treatment with OA involves significant morbidity and carries risk of undesired consequences, such as frozen abdomen and enteroatmospheric fistulas [4–6]. Delayed primary fascial closure can mostly be achieved when utilizing dynamic fascial traction systems combined with negative pressure wound therapy (typically vacuum-assisted wound closure and mesh-mediated fascial traction, VAWCM) [7, 8]. Despite risk of serious consequences, OA treatment is potentially life-saving when patient endures worsening organ dysfunction due to treatment-resistant ACS [9, 10].

As organ failure is characteristical and ACS occurs commonly in SAP, patient selection and correct timing of OA treatment remains clinically challenging. Enlightening what influences the decision to engage OA treatment might help narrow down selection of patients for this morbid treatment. The main aim of this study was to identify risk factors associated with the choice of OA treatment in patients with SAP by comparing patients with SAP that underwent OA treatment with matched control patients with SAP that were managed conservatively. Secondarily, we report and compare the outcomes of these patients.

## Methods

This was a matched case-control study of patients with SAP comparing those that underwent OA treatment with conservatively treated matched controls.

### Data collection

We searched Helsinki University Hospital patient database to obtain all patients with acute pancreatitis treated between September 1, 2009 and December 31, 2019, at Meilahti Hospital intensive care unit (ICU). Investigator (H.H.) screened electronic medical records for possible OA treatment of all patients with discharge ICD-10 diagnosis code K85.X or K86.X from ICU within mentioned timeframe. Study included only patients with SAP according to the Revised Atlanta Classification [11]. Exclusion from study occurred if admission to ICU was later than 10 days or OA treatment later than 4 weeks after hospital admission. In addition, initial treatment abroad or pancreas transplant pancreatitis excluded patient from study. We collected a pre-specified list of variables and summoned the information into a separate patient database, replacing patient identification information with running numbering. We collected the worst and the best values of physiological parameters, such as IAP, at 12-h intervals, and for laboratory test results at daily intervals. These variables were collected preceding OA treatment initiation for OA group patients and for the corresponding time for matched control group patients.

Conduction of study adhered to STROBE-guidelines (https://strobe-statement.org/). Department of Abdominal Surgery at Helsinki University Hospital approved conduction of study. Institutional ethical committee assessment was not required.

### Definitions

Intra-abdominal hypertension was defined according to consensus guidelines [4]. Repeatedly elevated IAP of more than 20 mmHg accompanied by a new or worsening of an existing organ failure defined as ACS [4]. In attempt to quantify exposure to possible intra-abdominal ischemia due to ACS, we summated the gross time of IAP higher than 20 mmHg accompanied by new or worsened existing organ failure. Ischemia was defined as irreversible necrosis of a viscera.

### Procedure

All patients in the OA group underwent midline laparotomy. We utilized vacuum-assisted wound closure and mesh-mediated fascial traction (VAWCM) for maintenance and delayed primary fascia closure attempt of OA [12]. Change of VAWCM with gradual approximation of fascial edges occurred with a 2-4 days interval in the operating theater. Selected patients had initially temporary static laparostomy closure (i.e., skin bridging Bogota bag), planning to continue with VAWCM treatment in subsequent reoperations. Examples of such patients were patients needing early second look (within 24 h) and patients needing continuous visual monitoring of intra-abdominal status due to imminent risk of ischemia or bleeding. We attempted delayed primary fascial closure in all cases when possible after ACS subsided and only used when needed anterior component separation technique via small separate horizontal transverse skin incisions. Skin grafting was used selectively for complexity class 3-4 OA with intention to later reconstruct symptomatic ventral hernia [4].

### Matching

Patients that underwent OA treatment were matched with controls in a 1:1 ratio. Control patients had SAP but avoided OA treatment within entire hospital treatment period. We calculated maximum daily Sequential Organ Failure Assessment (SOFA) score for each patient in OA group and possible matched control during the first 3 days after ICU admission. The primary matching criteria was highest SOFA score within 72 h from ICU admission. When there was more than one eligible control patient, we used (in order of importance) age, preceding comorbidities, and year of treatment as secondary matching criteria. Maximum deviance of SOFA score ± 2 and age ± 10 years were allowed between OA-treated patients and their matched controls.

### Statistical analysis

We analyzed the acquired data in SPSS (IBM. Corp., Armonk, NY, USA). Level of statistical significance was *P* < 0.05. Fisher’s exact two-sided test, Mann-Whitney U test, and log-rank test were used as appropriate. Receiver operating characteristics (ROC) analysis guided conversion of continuous variables to dichotomous.

For the primary aim, we compared mean and most abnormal values of physiological parameters in OA and control group patients by means of univariate analysis. Clinically interesting variables with statistical significance in univariate analysis that had a meaningful cut-off value in ROC analysis were entered into backward logistic multivariable analysis. We calculated odds ratios (OR) and 95% confidence intervals (CI) for the multivariable logistic regression analysis.

For the secondary aim, we compared outcomes of OA and matched control group patients by means of univariate analysis. We performed a post hoc univariate analysis of potential predictors of visceral ischemia in OA group patients.

## Results

### Matching

**Fig. 1:**
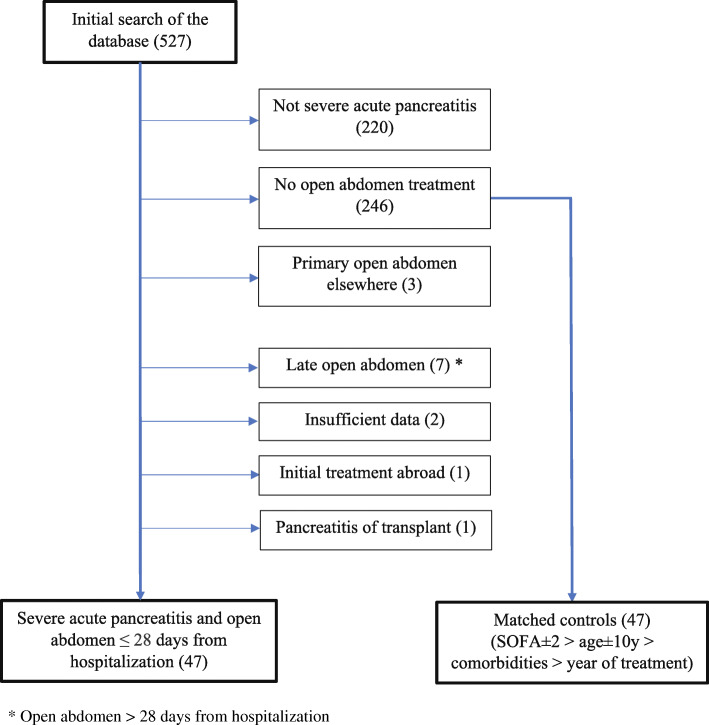
Flow chart

The systematic search of patient database resulted in 47 patients with OA treatment matched with 47 conservatively treated controls. Flow chart (Fig. 1) illustrates patient obtainment in study. Additional file 1 provides detailed information of patient matching. Due to otherwise insufficient number of control patients, we allowed a violation of the pre-specified matching principles in the following three cases: one OA-treated patient with SOFA score 19 was matched with a control patient with SOFA score 16, and two patients were matched despite 11 years age difference.

### Patients

Baseline characteristics of patients appear in Table 1. Forty (85%) patients in OA group suffered from ACS compared to 21 (45%) control patients, *P* <0.001. In OA group, 32 (68%) patients suffered from less than 20 ml/h urine output compared to 14 (30%) patients in control group, *P* < 0.001. Indication for OA treatment was refractory ACS in 40 (85%) patients, and abdomen was left open after explorative laparotomy in 7 (15%) patients. OA treatment commenced at a median of 60 (IQR 36-175) and 37 (IQR 17-125) hours from admission to hospital and ICU, respectively.

**Table 1:** Patient characteristics at admission to intensive care unit

	Open abdomen (n = 47)	Matched Controls (n = 47)	*P*
Age, median (range), years	49 (27–82)	50 (18–78)	0.456
BMI, mean ± SD, kg/m^2^	30.4 ± 4.9	30.4 ± 4.9	0.991
Male sex	42 (89)	41 (87)	1.000
Alcoholic etiology *	40 (85)	34 (72)	0.207
Tertiary referral	18 (38)	16 (34)	0.830
ASA ≥ III	32 (68)	25 (53)	0.205
SOFA < 72 h ^†^	13 (11–14)	12 (10–15)	0.876
APACHE II < 24 h ^†^	23 (17–29)	18 (15–26)	0.056
Time interval between hospital and ICU admission, hours ^†^	23 (8–33)	28 (12–40)	0.318

Presented values are absolute number of patients (number in brackets is percentage) unless stated otherwise

*Open abdomen: biliary (2), hypertriglyceridemia (2), other/idiopathic (2), postoperative (1). Matched controls: biliary (7), other/idiopathic (4), hypertriglyceridemia (1), post-ERCP (1)

^†^Median (interquartile range)

*APACHE II*, Acute Physiology and Chronic Health Evaluation II Scoring System; *ASA*, American Society of Anesthesiologists Physical Status Classification System; *BMI*, body mass index; *CI*, confidence interval; *ERCP*, endoscopic retrograde cholangiopancreatography; *ICU*, intensive care unit; *OR*, odds ratio; *SD*, standard deviation; *SOFA*, Sequential Organ Failure Assessment score

### Factors associated with OA treatment

Shown in Table 2 are results of univariate analysis of physiological parameters in OA group patients and matched controls. Rationale behind conversion of continuous to dichotomous variables is shown in Additional file 2. Table 3 summons the results of multivariable analysis of factors associated with OA treatment. Less than 20 ml/h urine output (OR 5.0 95% CI 1.8-13.7) and ACS (OR 4.6 95% CI 1.4-15.2) independently associated with increased risk of OA treatment.

**Table 2:** Univariate analysis of physiological parameters in open abdomen and matched control group

	Open abdomen (n = 47)	Matched controls (n = 47)	*P*
MAP lowest (24 h) ± SD, mmHg *	63 ± 9	67 ± 10	0.092
MAP mean ± SD, mmHg ^†^	79 ± 8	85 ± 11	**0.004**
IAP highest (24 h), ± SD, mmHg *^‡^	24 ± 4	21 ± 5	**< 0.001**
IAP mean ± SD, mmHg ^†‡^	20 ± 3	17 ± 3	**< 0.001**
APP lowest (24 h), ± SD, mmHg *^‡^	46 ± 9	56 ± 13	**< 0.001**
APP mean ± SD, mmHg ^†‡^	58 ± 9	68 ± 11	**< 0.001**
Urine output lowest (24 h) ± SD, ml/h *	19 ± 29	41 ± 36	**< 0.001**
Urine output mean ± SD, ml/h ^†^	31 ± 31	72 ± 66	**< 0.001**
Plasma creatinine highest (24 h) ± SD, umol/L *	216 ± 163	213 ± 174	0.484
Plasma creatinine mean ± SD, umol/L ^†^	209 ± 159	181 ±146	0.064
Plasma urea highest (24 h) ± SD, mmol/L *^‡^	11.9 ± 9.0	13.2 ± 10.2	0.692
Plasma urea mean ± SD, mmol/L ^†‡^	11.3 ± 8.5	11.3 ± 7.8	0.753
PaO2/FiO2 lowest (24 h) ± SD *	142 ± 58	140 ± 66	0.631
PaO2/FiO2 mean ± SD †	199 ± 65	207 ± 76	0.973
Blood leucocyte count highest (24 h) ± SD, 10^9^/L	18.7 ± 12.9	14.1 ± 7.8	0.119
Blood leucocyte count mean ± SD, 10^9^/L	16.0 ± 9.1	11.9 ± 6.1	**0.016**
Platelet count lowest (24 h) ± SD, 10^9^/L *	162 ± 122	120 ± 105	0.083
Platelet count mean ± SD, 10^9^/L ^†^	142 ± 83	142 ± 114	0.666
Plasma bilirubin highest (24 h) ± SD, umol/L *	45 ± 46	58 ± 51	**0.041**
Plasma bilirubin mean ± SD, umol/L ^†^	43 ± 41	48 ± 38	0.261
Plasma CRP highest (24 h) ± SD, mg/L *	319 ± 129	319 ± 170	0.725
Plasma CRP mean ± SD, mg/L ^†^	311 ± 128	273 ± 134	0.150
GCS lowest (24 h) (IQR) *	15 (13–15)	15 (14–15)	0.509
GCS mean (IQR) ^†^	15 (14–15)	15 (14–15)	0.672
Plasma lactate highest (24 h) ± SD, mmol/L *	4.8 ± 4.2	3.5 ± 2.6	0.403
Plasma lactate mean ± SD, mmol/L ^†^	4.5 ± 3.9	2.9 ± 2.6	**0.014**
Base-excess lowest (24 h) ± SD, mmol/L *	−8.0 ± 6.6	−7.6 ± 4.2	0.871
Base-excess mean ± SD, mmol/L ^†^	−7.5 ± 6.4	−5.1 ± 4.6	0.085
Arterial pH, mean ± SD ||	7.22 ± 0.14	7.29 ± 0.10	**0.016**
Serum potassium, mean ± SD, mmol/l ||	5.3 ± 1.1	4.6 ± 0.9	**0.001**
Serum sodium, mean ± SD, mmol/l ||	127 ± 5	130 ± 6	**0.008**
Cumulative excess fluid balance ± SD, ml	12267 ± 7374	8616 ± 6888	**0.020**

*Mean of most divergent value within 24 h from laparostomy (OA group) vs. mean of most divergent value within entire follow-up period (matched controls)

†Mean of all preceding values

‡One missing value in group open abdomen (n = 46)

||Mean of most divergent values within 24 h from ICU admission

*APP*, abdominal perfusion pressure; *CI*, confidence interval; *CRP*, C-reactive protein; *GCS*, Glasgow coma scale; *IAP*, intra-abdominal pressure; *IQR*, interquartile range; *MAP*, mean arterial pressure; *OR*, odds ratio; *SD*, standard deviation

**Table 3:** Multivariable analysis of factors associated with open abdomen

	OR (95% CI)	*P*
Urine output ≤ 20 ml/h *	4.99 (1.82–13.69)	**0.002**
Abdominal compartment syndrome ^†^	4.64 (1.42–15.20)	**0.011**
IAP ≥ 24 mmHg *	3.00 (0.98–9.20)	0.055

Backward conditional logistic regression based on data in Table 2. Variables for the model were chosen based on clinical usefulness and statistical significance. Receiver operator characteristics curve was plotted to convert continuous variables to dichotomous. Other variables that were included in model: APP < 50 mmHg*, cumulative excess fluid balance > 10,000 ml.

*Most divergent value within 24 h from laparostomy for OA group and within entire follow-up period for matched controls

†IAP > 20 mmHg and new or worsening of existing organ failure

*CI*, confidence interval; *IAP*, intra-abdominal pressure; *OR*, odds ratio; *SD*, standard deviation

### Comparison of outcomes

As can be seen in Table 4, OA group patients suffered significantly more often than matched controls from visceral ischemia (16 [34%] vs 3 [6%] patients, *P* = 0.002), needed more often necrosectomy (26 [55%] vs 10 [21%] patients, *P* = 0.001), and overall mortality was higher (20 [42%] vs 8 [17%] patients, *P* = 0.010). In 90-day survivors, overall median hospital (73 vs 30 days) and ICU (37 vs 14 days) lengths of stays were longer in OA group than control patients, *P* <0.001.

**Table 4:** Univariate analysis of outcomes in open abdomen and matched control group

	Open abdomen (n = 47)	Matched controls (n = 47)	*P*
Mortality within 90 days from ICU admission			
All patients	20 (43)	8 (17)	**0.010**
Patients with visceral ischemia *	10 (63)	2 (67)	0.685
Patients without visceral ischemia *	10 (32)	6 (14)	0.066
Patients with abdominal compartment syndrome *^†^	19 (48)	3 (14)	**0.013**
Visceral ischemia	16 (34)	3 (6)	**0.002**
Necrosectomy within 90 days from ICU admission	26 (55)	10 (21)	**0.001**
Survivor without necrosectomy ‡	9 (19)	30 (64)	**0.004**
Time interval between ICU admission and death, median (range), days	13 (0-73)	1 (0-88)	0.138

Number in brackets means percentage unless stated otherwise. Mortality/survival analysis implemented log-rank test. Remaining analysis utilized Fisher’s exact 2-sided test

*Presented is the percentage of patients with the risk factor in question

†Forty open abdomen patients and 21 matched controls suffered from abdominal compartment syndrome

‡Patients that did not undergo necrosectomy and survived 90 days following ICU admission

*ICU*, intensive care unit

### Visceral ischemia

In the OA group, median time of ACS duration before initiating OA treatment was statistically similar in patients with (median 7 h, IQR 1-38 h) and without (median 14 h, IQR 7-33h) visceral ischemia, *P* = 0.317. Altogether 11 (23%) OA-treated patients suffered from bowel ischemia, including colonic, small bowel, and both colonic and small bowel ischemia in 6 (13%), 1 (2%), and 4 (9%) patients, respectively. Independently or in conjunction with bowel ischemia, 7 (15%), 3 (6%), and 1 (2%) OA-treated patient suffered from gall bladder, omental, and gastric ventricle ischemia, respectively. Three (6%) patients from matched control group suffered from colonic ischemia, and small bowel ischemia appeared jointly in all but one. Two of these patients had endured ACS and the third patient had acute renal failure and needed continuous renal replacement therapy. Overall mortality of 19 patients with visceral ischemia (including patients from both study groups) was 63%, including 6 deaths out of 7 patients with small bowel ischemia. Post hoc analysis of risk factors for visceral ischemia in OA-treated patients only found lower mean CRP value in patients enduring ischemia compared to other patients (Additional file 3). Presented in Additional file 4 are detailed description of all patients with visceral ischemia.

### Abdominal closure

Thirteen (28%) patients died with ongoing OA treatment. Among 34 patient who survived to abdominal closure, 33 (97%) had delayed primary fascial closure (including 5 [15%] patients requiring separation of components), and one (3%) patient needed split-thickness skin grafting on granulated abdominal wound due to frozen abdomen and enteroatmospheric fistula. Median time interval between initiated OA treatment and abdominal closure was 20 (IQR 12-28) days.

## Discussion

We conducted a study comparing OA- and conservatively treated SAP patients. Results show that decreased urine output and ACS were independently associated with the choice of OA treatment in SAP. Ischemia was remarkably common in OA-treated patients, but conventional physiological parameters were inaccurate predictors of developing ischemia. As expected, OA treatment associated with increased morbidity and mortality, most probably due to a more severe disease than the decompressive surgery itself. Delayed primary fascial closure was almost always achievable if patients endured and survived the OA treatment period.

Present study found an independent association between decreased urine output and utilization of OA treatment in SAP. In line with such clinical practice, a recent meta-analysis of patients with ACS for various reasons concluded that especially urine output alongside respiratory parameters improve following OA treatment [13]. In an experimental animal model of ACS in SAP, reduction of urine output was reversible to baseline if laparostomy was performed before urine output decreased to around 20 ml/h [14]. Urine output declining to 20 ml/h (oliguria) independently predicted choice of OA treatment in our study; however, this result cannot be interpreted as a guideline for initiating OA treatment as such. A significant number of control patients were treated conservatively despite declining urine output. There is need for further research to determine a potentially optimal threshold for decompression in SAP when ACS is persistent.

Since refractory and treatment resistant ACS is considered an indication for OA treatment, it is intuitive that current study showed an independent association between existence of ACS and subsequent choice of OA treatment. Interestingly, around half of control group patients suffered from ACS, and endured ACS that lasted for at least as long as in OA group patients. Although not quantifiable with the current studied variables, it is likely that patients selected for OA treatment simply were deteriorating faster with an illness more resistant to maximal supportive treatment. We find that the substantial proportion of patients with visceral ischemia symbolizes the distress that led to intervention.

Although presented in sophisticated animal models [15, 16], to date, no study in humans has proven correlation between elevation of IAH and development of visceral ischemia. The available data regarding ischemic complications in SAP is sparse. Smit et al. reported higher incidence (61.5%) of intra-abdominal ischemia in a series of 13 SAP-patients with ACS [17]. In study by Maatman and colleague’s, ischemia or perforation of the colon was present in around 7%; however, this cohort included the entire spectrum of patients with necrotizing pancreatitis. In a recent systematic review of patients with ACS of multiple etiologies, cause of death was related to intestinal ischemia in 15% of patients [13]. A third of OA-treated patients in the present study suffered from visceral ischemia despite decompressive effort. For these patients, decompression might have come too late, as irreversible development of ischemia had already occurred. Another possible explanation is that ischemia developed due to visceral hypoperfusion unrelated to intra-abdominal pressure, which has previously been associated with a similarly poor outcome as in patients with visceral ischemia in our study [18]. Only a third of our study patients with visceral ischemia survived, which is comparable to what has previously been reported (55%) [17]. Based on the current experience, small bowel ischemia within the context of SAP associates with a devastating outcome, as mortality reached nearly 90%. We were, unfortunately, unable to find meaningful clinical parameters that would have predicted evolving ischemia.

In the present study, around half of patients with OA treatment for ACS died within 90 days. Risk of death is around two-thirds when considering only patients with visceral ischemia. These outcomes are in line with previously reported mortality between 25 and 71% in patients with laparostomy for ACS in SAP [9, 17, 19, 20]. As shown by our results, the remainder of patients treated with OA suffers from significant morbidity in terms of repeat invasive procedures and length of stay. If patient survives past the initial struggles and intra-abdominal conditions become more favorable, delayed fascial closure of abdomen is achievable in almost all patients. This result is comparable with previous experiences of VAWCM treatment in patients with OA [21]. Despite past decades increased knowledge in prevention and effects of elevated IAP in the critically ill, the outcomes of our study patients are generally quite upsetting, especially as maximal invasive treatment efforts were invested. This study cannot provide any estimate of what effect OA treatment might have on outcomes as underlying physiological derangements are likely to be different between groups. The reported associations on outcomes should therefore not be interpreted as causation.

Our results show that ACS is a common finding in patients with SAP, and that far from all patients requires surgical decompression despite persistence to conservative management. Ischemia is a common finding following laparostomy for ACS in SAP. Unfortunately, efforts to salvage patient were mostly futile when ischemia had occurred. Irreversible ischemic changes manifested in these patients previous to laparostomy. Unfortunately, no conclusions can be made whether earlier intervention might have improved the outcome.

An obvious weakness of this study is the observational retrospective nature. Matching principles is a known potential introducer of unsolicited selection bias. Despite our efforts to find conservatively treated matching peers by early phase maximal initial SOFA score, it seems that OA-treated patients still were sicker at baseline. Difference in APACHE II score at admission to ICU depicts this trend; however, choice of a different risk stratification tool than SOFA score would likely not have obviated this selection bias. Due to the rarity of patients even being considered for surgical decompression of ACS in SAP, there are to date no randomized trials comparing conservative and OA treatment, which depicts the difficulty to study such events. Limiting the applicability of our study results is that indication for OA treatment was not standardized and depended on shared decision-making by the surgeon on call and anesthesiologist in charge of patient at ICU. Another weakness is the absence of standardized long-time follow-up that could have shed light on associated long-time morbidity, functional outcomes, and quality of life.

## Conclusions

In patients with SAP, decreased urine output under 20 ml/h and fulfilling ACS criteria independently increase the risk of OA treatment. OA-treated patients often suffer from visceral ischemia; however, clinically meaningful predictors of ischemia seem hard to identify.

## Supplementary Information

**Additional file 1.** : Open Abdomen Patients (Case) and Their Matched Controls (Control)

**Additional file 2.** : ROC analysis of Continuous Variables (from Table 2)

**Additional file 3.** : Univariate Analysis of Physiological Parameters Preceding Visceral Ischemia in Patients with Open Abdomen

**Additional file 4.** : Characteristics of Patients with Severe Acute Pancreatitis and Visceral Ischemia

## Data Availability

We have not filed for permission to publish the study material.

## Abbreviations

ACS: Abdominal compartment syndrome
IAH: Intra-abdominal hypertension
IAP: Intra-abdominal pressure
OA: Open abdomen
ROC: Receiver operating characteristics
SAP: Severe acute pancreatitis
VAWCM: Vacuum-assisted wound closure and mesh-mediated fascial traction

## References

[1] Aitken EL, Gough V, Jones A, Macdonald A (2014). Observational study of intra-abdominal pressure monitoring in acute pancreatitis. Surgery.

[2] De Waele JJ, Leppäniemi AK (2009). Intra-abdominal hypertension in acute pancreatitis. World J Surg.

[3] van Brunschot S, Schut AJ, Bouwense SA, Besselink MG, Bakker OJ, van Goor H, Hofker S, Gooszen HG, Boermeester MA, van Santvoort H, Dutch Pancreatitis Study Group (2014). Abdominal compartment syndrome in acute pancreatitis: a systematic review. Pancreas.

[4] Kirkpatrick AW, Roberts DJ, De Waele J (2013). Intra-abdominal hypertension and the abdominal compartment syndrome: updated consensus definitions and clinical practice guidelines from the World Society of the Abdominal Compartment Syndrome. Intensive Care Med.

[5] Coccolini F, Roberts D, Ansaloni L, Ivatury R, Gamberini E, Kluger Y, Moore EE, Coimbra R, Kirkpatrick AW, Pereira BM, Montori G, Ceresoli M, Abu-Zidan FM, Sartelli M, Velmahos G, Fraga GP, Leppaniemi A, Tolonen M, Galante J, Razek T, Maier R, Bala M, Sakakushev B, Khokha V, Malbrain M, Agnoletti V, Peitzman A, Demetrashvili Z, Sugrue M, di Saverio S, Martzi I, Soreide K, Biffl W, Ferrada P, Parry N, Montravers P, Melotti RM, Salvetti F, Valetti TM, Scalea T, Chiara O, Cimbanassi S, Kashuk JL, Larrea M, Hernandez JAM, Lin HF, Chirica M, Arvieux C, Bing C, Horer T, de Simone B, Masiakos P, Reva V, DeAngelis N, Kike K, Balogh ZJ, Fugazzola P, Tomasoni M, Latifi R, Naidoo N, Weber D, Handolin L, Inaba K, Hecker A, Kuo-Ching Y, Ordoñez CA, Rizoli S, Gomes CA, de Moya M, Wani I, Mefire AC, Boffard K, Napolitano L, Catena F (2018). The open abdomen in trauma and non-trauma patients: WSES guidelines. World J Emerg Surg.

[6] Cirocchi R, Birindelli A, Biffl WL, Mutafchiyski V, Popivanov G, Chiara O, Tugnoli G, di Saverio S (2016). What is the effectiveness of the negative pressure wound therapy (NPWT) in patients treated with open abdomen technique? A systematic review and meta-analysis. J Trauma Acute Care Surg.

[7] Poortmans N, Berrevoet F (2020). Dynamic closure techniques for treatment of an open abdomen: an update. Hernia.

[8] López-Cano M, García-Alamino JM, Antoniou SA, Bennet D, Dietz UA, Ferreira F, Fortelny RH, Hernandez-Granados P, Miserez M, Montgomery A, Morales-Conde S, Muysoms F, Pereira JA, Schwab R, Slater N, Vanlander A, van Ramshorst GH, Berrevoet F (2018). EHS clinical guidelines on the management of the abdominal wall in the context of the open or burst abdomen. Hernia.

[9] Mentula P, Hienonen P, Kemppainen E, Puolakkainen P, Leppäniemi A (2010). Surgical decompression for abdominal compartment syndrome in severe acute pancreatitis. Arch Surg.

[10] Leppäniemi A, Tolonen M, Tarasconi A, Segovia-Lohse H, Gamberini E, Kirkpatrick AW, Ball CG, Parry N, Sartelli M, Wolbrink D, van Goor H, Baiocchi G, Ansaloni L, Biffl W, Coccolini F, di Saverio S, Kluger Y, Moore E, Catena F (2019). 2019 WSES guidelines for the management of severe acute pancreatitis. World J Emerg Surg.

[11] Banks PA, Bollen TL, Dervenis C, Gooszen HG, Johnson CD, Sarr MG, Tsiotos GG, Vege SS, Acute Pancreatitis Classification Working Group (2013). Classification of acute pancreatitis--2012: revision of the Atlanta classification and definitions by international consensus. Gut.

[12] Petersson U, Acosta S, Björck M (2007). Vacuum-assisted wound closure and mesh-mediated fascial traction--a novel technique for late closure of the open abdomen. World J Surg.

[13] Van Damme L, De Waele JJ (2018). Effect of decompressive laparotomy on organ function in patients with abdominal compartment syndrome: a systematic review and meta-analysis. Crit Care.

[14] Ke L, Ni H, Tong Z (2013). The importance of timing of decompression in severe acute pancreatitis combined with abdominal compartment syndrome. J Trauma Acute Care Surg.

[15] Diebel LN, Dulchavsky SA, Brown WJ (1997). Splanchnic ischemia and bacterial translocation in the abdominal compartment syndrome. J Trauma.

[16] Cheng J, Wei Z, Liu X, Li X, Yuan Z, Zheng J, Chen X, Xiao G, Li X (2013). The role of intestinal mucosa injury induced by intra-abdominal hypertension in the development of abdominal compartment syndrome and multiple organ dysfunction syndrome. Crit Care.

[17] Smit M, Buddingh KT, Bosma B, Nieuwenhuijs VB, Hofker HS, Zijlstra JG (2016). Abdominal compartment syndrome and intra-abdominal ischemia in patients with severe acute pancreatitis. World J Surg.

[18] Hirota M, Inoue K, Kimura Y, Mizumoto T, Kuwata K, Ohmuraya M, Ishiko T, Beppu T, Ogawa M (2003). Non-occlusive mesenteric ischemia and its associated intestinal gangrene in acute pancreatitis. Pancreatology.

[19] Davis PJB, Eltawil KM, Abu-Wasel B, Walsh MJ, Topp T, Molinari M (2013). Effect of obesity and decompressive laparotomy on mortality in acute pancreatitis requiring intensive care unit admission. World J Surg.

[20] Ke L, Ni H, Sun J, Tong ZH, Li WQ, Li N, Li JS (2012). Risk factors and outcome of intra-abdominal hypertension in patients with severe acute pancreatitis. World J Surg.

[21] Rasilainen S, Mentula P, Salminen P, Koivukangas V, Hyöty M, Mäntymäki LM, Pinta T, Haikonen J, Rintala J, Rantanen T, Strander T, Leppäniemi A (2020). Superior primary fascial closure rate and lower mortality after open abdomen using negative pressure wound therapy with continuous fascial traction. J Trauma Acute Care Surg.

