# Supplementary material for: Who would benefit from open abdomen in severe acute pancreatitis?—a matched case-control study
Source: World J Emerg Surg. 2021 Jun 10;16:32. doi: 10.1186/s13017-021-00376-x (PMC8194042; doi:10.1186/s13017-021-00376-x)
Supplement: Supplementary file 1 — Additional file 1. : Open Abdomen Patients (Case) and Their Matched Controls (Control) [file 13017_2021_376_MOESM1_ESM.pdf]

Additional file 1. Open Abdomen Patients (Case) and Their Matched Controls (Control)

ID	Group	Pair ID	SOFA <72h	Age	Comorbidities	Year
1	Case	101	20	56	Yes	2019
101	Control	1	18	61	Yes	2017
2	Case	102	19	27	Yes	2017
102	Control	2	17	34	No	2010
3	Case	103	**19**	33	Yes	2014
103	Control	3	**16**	38	Yes	2018
4	Case	104	17	39	Yes	2015
104	Control	4	15	44	No	2015
5	Case	105	16	32	No	2010
105	Control	5	15	33	No	2011
6	Case	106	16	**49**	No	2018
106	Control	6	17	**60**	Yes	2015
7	Case	107	16	61	Yes	2016
107	Control	7	18	70	No	2017
8	Case	108	16	70	No	2018
108	Control	8	17	77	Yes	2013
9	Case	109	15	49	No	2012
109	Control	9	15	50	Yes	2012
10	Case	110	14	43	No	2019
110	Control	10	15	47	No	2013
11	Case	111	14	38	No	2014
111	Control	11	14	33	No	2010
12	Case	112	14	52	No	2014
112	Control	12	14	51	No	2017
13	Case	113	14	**56**	No	2015
113	Control	13	15	**67**	No	2011
14	Case	114	14	62	No	2011
114	Control	14	16	72	No	2012
15	Case	115	13	32	No	2010
115	Control	15	12	35	Yes	2013
16	Case	116	13	32	No	2015
116	Control	16	11	36	No	2012
17	Case	117	13	41	No	2013
117	Control	17	14	44	No	2012
18	Case	118	13	45	Yes	2015
118	Control	18	13	47	Yes	2017
19	Case	119	13	46	No	2016
119	Control	19	13	48	Yes	2014
20	Case	120	13	49	No	2019
120	Control	20	14	55	Yes	2016
21	Case	121	13	53	No	2015
121	Control	21	12	53	No	2018
22	Case	122	13	53	Yes	2016
122	Control	22	12	52	Yes	2017
23	Case	123	13	55	No	2017
123	Control	23	13	59	Yes	2010
24	Case	124	13	55	No	2019
124	Control	24	13	62	No	2014

Additional file 1. Open Abdomen Patients (Case) and Their Matched Controls (Control)

ID	Group	Pair ID	SOFA <72h	Age	Comorbidities	Year
25	Case	125	12	34	No	2014
125	Control	25	11	40	No	2012
26	Case	126	12	44	No	2010
126	Control	26	12	40	No	2017
27	Case	127	12	51	No	2011
127	Control	27	12	46	No	2010
28	Case	128	12	54	No	2018
128	Control	28	12	62	No	2012
29	Case	129	12	61	No	2016
129	Control	29	12	71	Yes	2010
30	Case	130	12	82	Yes	2012
130	Control	30	13	78	Yes	2011
31	Case	131	11	30	No	2013
131	Control	31	12	21	No	2015
32	Case	132	11	38	No	2015
132	Control	32	11	40	No	2017
33	Case	133	11	50	Yes	2009
133	Control	33	11	51	No	2018
34	Case	134	11	53	No	2015
134	Control	34	10	53	No	2014
35	Case	135	11	57	Yes	2012
135	Control	35	11	58	Yes	2018
36	Case	136	11	63	Yes	2010
136	Control	36	10	60	No	2017
37	Case	137	11	66	No	2015
137	Control	37	11	67	Yes	2017
38	Case	138	10	33	Yes	2011
138	Control	38	10	33	No	2017
39	Case	139	10	27	No	2012
139	Control	39	10	18	No	2014
40	Case	140	10	52	No	2014
140	Control	40	10	50	No	2018
41	Case	141	10	65	No	2013
141	Control	41	10	60	No	2017
42	Case	142	10	48	No	2011
142	Control	42	10	42	No	2015
43	Case	143	10	58	Yes	2013
143	Control	43	10	59	Yes	2018
44	Case	144	9	33	No	2018
144	Control	44	10	40	No	2013
45	Case	145	9	38	Yes	2012
145	Control	45	9	45	No	2012
46	Case	146	8	50	No	2017
146	Control	46	8	48	No	2012
47	Case	147	5	45	No	2013
147	Control	47	5	42	No	2017
