# Supplementary material for: Who would benefit from open abdomen in severe acute pancreatitis?—a matched case-control study
Source: World J Emerg Surg. 2021 Jun 10;16:32. doi: 10.1186/s13017-021-00376-x (PMC8194042; doi:10.1186/s13017-021-00376-x)
Supplement: Supplementary file 2 — Additional file 2. : ROC analysis of Continuous Variables (from Table 2) [file 13017_2021_376_MOESM2_ESM.pdf]

Additional file 2. ROC Analysis of Continuous Variables (from Table 2)

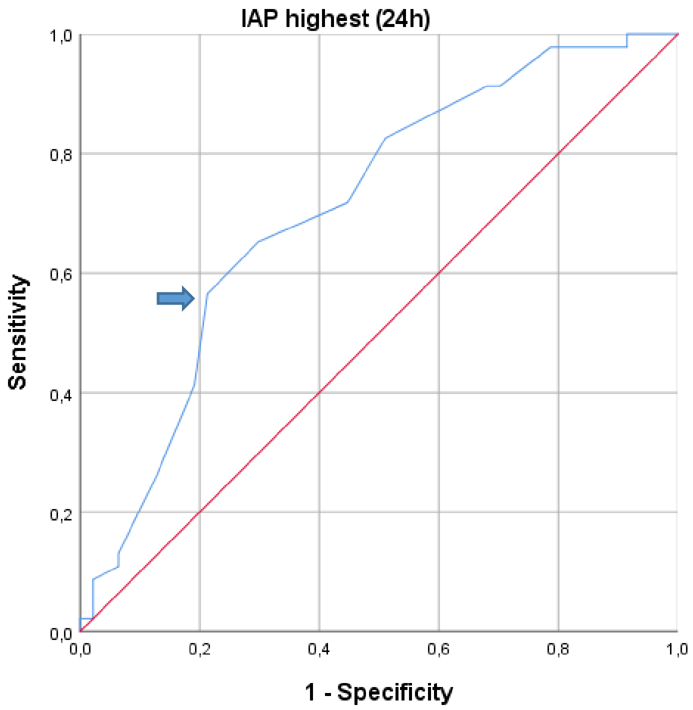

AUROC 0.710 (95%CI 0.604-0.816), Arrow: IAP ≥ 24 mmHg (Sensitivity 57%, Specificity 79%)

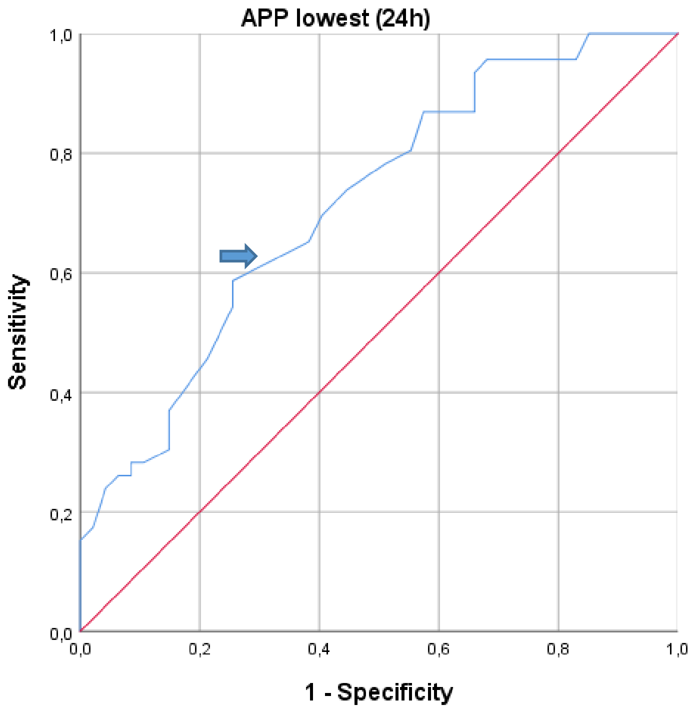

AUROC 0.715 (95%CI 0.612-0.818), Arrow: APP < 50 mmHg (Sensitivity 61%, Specificity 70%)

Additional file 2. ROC Analysis of Continuous Variables (from Table 2)

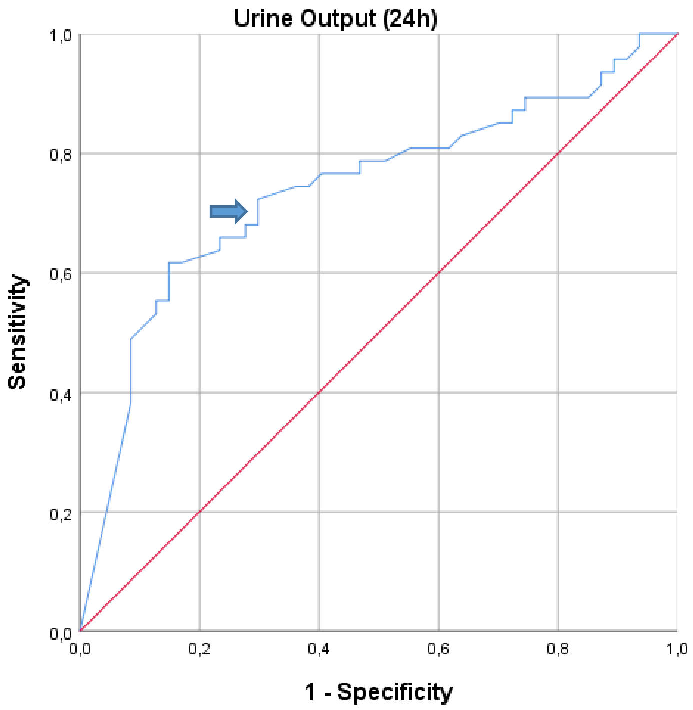

AUROC 0.739 (95%CI 0.636-0.843), Arrow: Urine output ≤ 20ml/h (Sensitivity 70%, Specificity 70%)

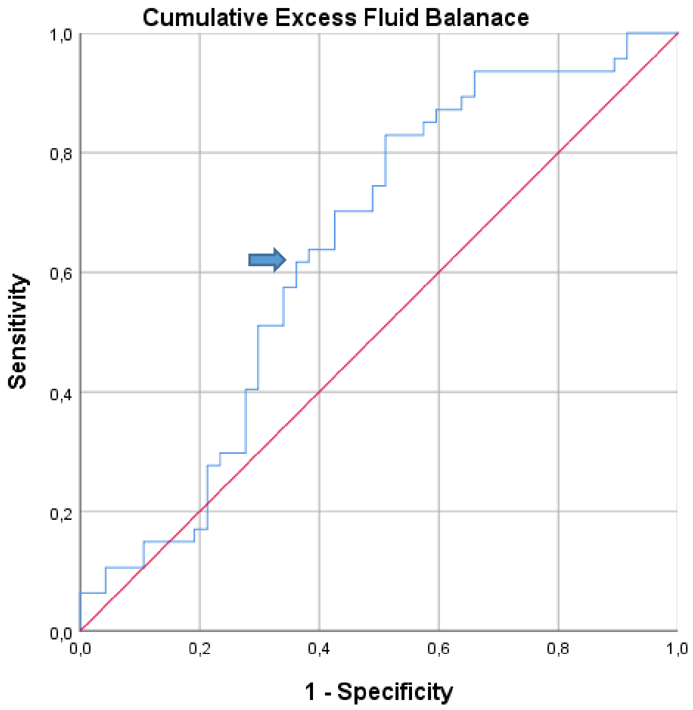

AUROC 0.640 (95%CI 0.526-0.754), Arrow: Cumulative Excess Fluid Balance > 10 000ml (Sensitivity 62%, Specificity 64%)
