# Supplementary material for: Who would benefit from open abdomen in severe acute pancreatitis?—a matched case-control study
Source: World J Emerg Surg. 2021 Jun 10;16:32. doi: 10.1186/s13017-021-00376-x (PMC8194042; doi:10.1186/s13017-021-00376-x)
Supplement: Supplementary file 3 — Additional file 3. : Univariate Analysis of Physiological Parameters Preceding Visceral Ischemia in Patients with Open Abdomen [file 13017_2021_376_MOESM3_ESM.pdf]

**Additional file 3. Univariate Analysis of Physiological Parameters Preceding Visceral Ischemia in Patients with Open Abdomen**			
	Ischemia (n=16)	No Ischemia (n=31)	*P*
Duration of ACS, mean, hours	7 (1-38)	14 (7-33)	
MAP lowest (24h) ± SD, mmHg *	64 ± 9	63 ± 10	0.645
MAP mean ± SD, mmHg †	78 ± 6	80 ± 9	0.271
IAP highest (24h), ± SD, mmHg *‡	23 ± 4	24 ± 4	0.346
IAP mean ± SD, mmHg †‡	20 ± 3	21 ± 3	0.467
APP lowest (24h), ± SD, mmHg *‡	47 ± 11	46 ± 8	0.606
APP mean ± SD, mmHg †‡	57 ± 9	58 ± 8	0.761
Urine output lowest (24h) ± SD, ml/h *	15 ± 28	20 ± 30	0.293
Urine output mean ± SD, ml/h †	27 ± 32	33 ± 31	0.323
Plasma creatinine highest (24h) ± SD, umol/L *	264 ± 242	192 ± 99	0.472
Plasma creatinine mean ± SD, umol/L †	258 ± 239	183 ± 91	0.351
Plasma urea highest (24h) ± SD, mmol/L *‖	16 ± 13	9.8 ± 4.7	0.065
Plasma urea mean ± SD, mmol/L †‖	15 ± 13	9.5 ± 3.9	0.163
PaO2/FiO2 lowest (24h) ± SD *	163 ± 55	131 ± 57	0.053
PaO2/FiO2 mean ± SD †	215 ± 57	191 ± 69	0.262
Blood leucocyte count highest (24h) ± SD, 10^9/L	21.3 ± 15.5	17.3 ± 11.4	0.486
Blood leucocyte count mean ± SD, 10^9/L	19.4 ± 12.0	14.2 ± 6.7	0.369
Platelet count lowest (24h) ± SD, 10^9/L *	135 ± 104	176 ± 130	0.234
Platelet count mean ± SD, 10^9/L †	115 ± 71	156 ± 86	0.133
Plasma bilirubin highest (24h) ± SD, umol/L *	45 ±53	45 ± 42	0.645
Plasma bilirubin mean ± SD, umol/L †	45 ± 49	43 ± 37	0.849
Plasma CRP highest (24h) ± SD, mg/L *	256 ± 120	352 ± 123	**0.025**
Plasma CRP mean ± SD, mg/L †	255 ± 125	340 ± 122	**0.029**
GCS lowest (24h) (IQR) *	15 (14-15)	15 (13-15)	0.549
GCS mean (IQR) †	15 (14-15)	15 (14-15)	0.361
Plasma lactate highest (24h) ± SD, mmol/L *	5.8 ± 4.0	4.4 ± 4.2	0.127
Plasma lactate mean ± SD, mmol/L †	5.3 ± 4.1	4.1 ± 3.9	0.178
Base-excess lowest (24h) ± SD, mmol/L *	-10.7 ± 7.0	-6.6 ± 6.0	0.094
Base-excess mean ± SD, mmol/L †	-9.5 ± 7.6	-6.4 ± 5.5	0.281
Arterial pH, mean ± SD §	7.20 ± 0.14	7.23 ± 0.14	0.212
Serum potassium, mean ± SD, mmol/l §	5.6 ± 1.2	5.2 ± 1.1	0.229
Serum sodium, mean ± SD, mmol/l §	126 ± 7	128 ± 5	0.299
Cumulative excess fluid balance ± SD, ml	14131 ± 7727	11242 ± 7172	0.069

* Mean of the most divergent values within 24h from laparostomy
† Mean of all preceding values
‡ One missing value in group Ischemia (n=15)
‖ One missing value in group No Ischemia (n=30)
§ Mean of most divergent values within 24h from ICU admission

ACS, Abdominal Compartment Syndrome; APP, Abdominal Perfusion Pressure; CI, Confidence Interval; CRP, C-Reactive Protein; GCS, Glasgow Coma Scale; IAP, Intra-abdominal Pressure; IQR, Interquartile Range; MAP, Mean Arterial Pressure; OR, Odds Ratio; SD, Standard Deviation
