# Supplementary material for: Who would benefit from open abdomen in severe acute pancreatitis?—a matched case-control study
Source: World J Emerg Surg. 2021 Jun 10;16:32. doi: 10.1186/s13017-021-00376-x (PMC8194042; doi:10.1186/s13017-021-00376-x)
Supplement: Supplementary file 4 — Additional file 4. : Characteristics of Patients with Severe Acute Pancreatitis and Visceral Ischemia [file 13017_2021_376_MOESM4_ESM.pdf]

Additional file 4. Characteristics of Patients with Severe Acute Pancreatitis and Visceral Ischemia										
Patient	Year	Day*	SOFA†	CRRT	ACS‡	Indication	Ischemia	Delay‖	OA	Outcome
27y male	2017	7	19	+	+	Worsening cardiovascular shock and respiratory insufficiency.	Gall bladder	0	+	Death
33y male	2014	15	16	+	+	Elevated lactate. Mechanical small bowel obstruction suspicion on CT scan.	Greater omentum	0	+	Death
49y male	2018	8	16	+	+	Worsening cardiovascular shock. Persistently elevated lactate and acidosis. Signs of gastrointestinal bleeding. Infected pancreatic necrosis on CT scan.	Gall bladder Gastric ventricle	1	+	Death
61y male	2016	0	16	+	+	Worsening cardiovascular shock. Persistently elevated lactate and acidosis.	Small intestine, Colon	0	+	Death
70y male	2018	2	16	+	?	Worsening MOF. Persistently elevated lactate. Diffuse intestinal oedema on CT scan. IAP not measured. Clinical suspicion of intestinal ischemia.	Small intestine, Colon	0	+	Death
43y male	2019	2	14	+	+	Worsening cardiovascular shock. Persistently elevated lactate and acidosis.	Gall bladder	1	+	Survived
62y male	2011	3	14	+	+	Worsening cardiovascular shock and respiratory insufficiency. Persistently elevated lactate and acidosis.	Small intestine, Colon	0	+	Death
65y male	2013	12	14	+	+	Worsening cardiovascular shock. Persistently elevated lactate and acidosis. Significant leucocytosis ($69x10^9$) and distended gall bladder on CT scan.	Gall Bladder, Colon	0	+	Death
32y male	2010	2	13	+	+	Worsening cardiovascular shock. Persistently elevated lactate and acidosis.	Gall bladder, Greater omentum, Colon	0	+	Death
41y female	2013	3	13	+	+	Worsening cardiovascular shock. Persistently elevated lactate and acidosis.	Small intestine Colon	0	+	Death
27y male	2012	7	12	+	+	Worsening cardiovascular shock.	Small intestine	0	+	Survived
61y female	2016	3	12	-	+	Declining urine output.	Gall bladder, Greater omentum, Colon	1	+	Death
50y male	2009	1	11	-	-	Suspicion of colon ischemia on CT scan.	Colon	0	+	Survived
48y male	2011	10	10	+	-	Suspicion of colon ischemia on CT scan.	Greater omentum, Colon	0	+	Survived
33y male	2018	7	9	-	-	Worsening MOF. Suspicion of colon ischemia on CT scan.	Colon	0	+	Survived
32y male	2015	14	7	-	+	Worsening cardiovascular shock. Persistently elevated lactate. Hematochezia. Ischemia rule-out.	Gall bladder	0	+	Survived
67y female	2011	15	20	+	-	**Matched Control.** Autopsy finding intestinal ischemia.	Small Intestine Colon		-	Death
62y female	2012	11	12	+	+	**Matched Control.** Autopsy finding intestinal ischemia.	Small intestine, Colon		-	Death
48y male	2012	10	10	-	+	**Matched Control.** Suspicion of perforated bowel (free intraperitoneal air) on CT scan.	Colon		-	Survived

Table presents characteristics of all patients experiencing visceral ischemia. Unless stated otherwise, these patients are from the Open Abdomen group.

* Time interval (days) between initial symptoms and diagnosis of visceral ischemia.
† **Open abdomen:** Highest SOFA score within 72h from OA (group OA). **Matched control:** Highest SOFA within 72h from laparotomy or death.
‡ **Open abdomen:** ACS preceding OA. **Matched control:** ACS preceding laparotomy or death.
‖ Time interval (days) between initial laparostomy and diagnosis of the first ischemic viscera

ACS, Abdominal Compartment Syndrome; CRRT, Continuous Renal Replacement Therapy; CT, Computed Tomography; IAP, Intra-abdominal Pressure; ICU, Intensive Care Unit; MOF, Multiple Organ Failure; OA, Open Abdomen; SI, Small Intestine; SOFA, Sequential Organ Failure Assessment score
